# The immunopathology of ANCA-associated vasculitis

**DOI:** 10.1007/s00281-014-0436-6

**Published:** 2014-07-24

**Authors:** Eoin F. McKinney, Lisa C. Willcocks, Verena Broecker, Kenneth G. C. Smith

**Affiliations:** 1 The Cambridge Institute for Medical Research and the Department of Medicine, University of Cambridge School of Clinical Medicine, Cambridge, UK; 2 The Vasculitis and Lupus Service, Addenbrooke’s Hospital, Cambridge, UK; 3 The Department of Pathology, Addenbrooke’s Hospital, Cambridge, UK

**Keywords:** ANCA, Vasculitis, Anti-neutrophil cytoplasmic antibody, PR3, MPO

## Abstract

The small-vessel vasculitides are a group of disorders characterised by variable patterns of small blood vessel inflammation producing a markedly heterogeneous clinical phenotype. While any vessel in any organ may be involved, distinct but often overlapping sets of clinical features have allowed the description of three subtypes associated with the presence of circulating anti-neutrophil cytoplasmic antibodies (ANCA), namely granulomatosis with polyangiitis (GPA, formerly known as Wegener’s Granulomatosis), microscopic polyangiitis (MPA) and eosinophilic granulomatosis with polyangiitis (eGPA, formerly known as Churg-Strauss syndrome). Together, these conditions are called the ANCA-associated vasculitidies (AAV). Both formal nomenclature and classification criteria for the syndromes have changed repeatedly since their description over 100 years ago and may conceivably do so again following recent reports showing distinct genetic associations of patients with detectable ANCA of distinct specificities. ANCA are not only useful in classifying the syndromes but substantial evidence implicates them in driving disease pathogenesis although the mechanism by which they develop and tolerance is broken remains controversial. Advances in our understanding of the pathogenesis of the syndromes have been accompanied by some progress in treatment, although much remains to be done to improve the chronic morbidity associated with the immunosuppression required for disease control.

The small-vessel vasculitides are a group of disorders characterised by inflammation of the walls of small blood vessels. The clinical phenotype is extremely heterogeneous and reflects the pattern of vessels affected by inflammation. While any vessel in any organ may be involved, distinct but often overlapping sets of clinical features have allowed the description of three subtypes predominantly affecting small calibre vessels, which are associated with both the presence of circulating anti-neutrophil cytoplasmic antibodies (ANCA) and by the lack of immune complex deposition. These are granulomatosis with polyangiitis (GPA, formerly known as Wegener’s Granulomatosis), microscopic polyangiitis (MPA) and eosinophilic granulomatosis with polyangiitis (eGPA, formerly known as Churg-Strauss syndrome). Together, these conditions are called the ANCA-associated vasculitidies (AAV). Other forms of non-ANCA-associated small-vessel vasculitis are typically characterised by either the presence of immune complex deposition (for example lupus vasculitis, Henoch-Schönlein purpura and Goodpasture’s disease) or a paraneoplastic phenomenon [1].

Many clinical features are common to all three types of AAV, including non-specific inflammatory symptoms such as malaise, fever, anaemia and weight loss, as well as organ specific involvement such as rash and synovitis. Differences in clinical features between conditions are inherently linked to the criteria used to define the conditions. Thus, GPA characteristically has ear, nose and throat (ENT) and/or respiratory involvement. Necrotising granulomata may cause sinusitis, nasal discharge, damage to the nasal septum, hearing loss and/or haemoptysis. eGPA is associated with asthma, eosinophilia and nasal polyps. MPA commonly affects the kidney without evidence of granulomata, upper respiratory tract involvement or asthma. The association with ANCA also varies: 80–90 % of individuals with GPA and MPA are ANCA positive, compared with 40 % of patients with eGPA (Churg-Strauss). The sensitivity for ANCA in the diagnosis of GPA (Wegener’s) and MPA is 81–85 %, whilst the specificity (if assayed by both immunofluorescence and ELISA) is 98 % [2]. The type of ANCA varies with AAV subtype; MPA is predominantly associated with antibodies to myeloperoxidase (MPO), whilst patients with GPA are more likely to have antibodies to proteinase 3 (PR3) [3]. Nonetheless, there is considerable overlap in the classification of AAV [3, 4].

## AAV: history

Wegener’s granulomatosis (WG) was arguably first described by a Scottish physician, McBride, who in 1897 reported a case of destructive inflammation of the nose and face which proved rapidly fatal and was pathologically distinct from infections known to produce similar features, such as syphilis, tuberculosis and ‘the glanders’ a zoonosis now known to be caused by Burkholderia mallei (infectious causes at that time being excluded by inoculation of live guinea pigs with purulent material) [5]. The first description of any necrotising vasculitis is attributed to Kussmaul and Meier [6], who described a systemic syndrome including kidney and nervous system involvement in 1866, coining the term ‘periarteritis nodosa’ which persists today in a revised form (as the increasingly rare vasculitis of medium-sized vessels, polyarteritis nodosa). Two further reports, one by Wohlwill in 1923 [7] of vascular inflammation akin to that seen in what is now termed microscopic polyangiitis (MPA) and a second by Klinger of granulomatous respiratory tract inflammation with necrotising glomerulonephritis in 1931 [8], both precede Friedrich Wegener’s graduation from medical school in 1932 [9]. Indeed, Wegener himself did not claim the eponym and was reportedly not keen to use it [10]. Latterly, a movement proposing that the eponymous title be dropped has developed following emergence of evidence linking Wegener to the Nazi regime during World War II [11]. It can then be noted with some irony that it was a Russian Jew, Jacob Churg (best-known for his description of the related AAV Churg-Strauss syndrome), who, along with Godman and after forced emigration to New York city during World War II, first attributed the condition to Wegener in 1954 [12]. Wegener’s work during the 1930s described a series of cases in detail [13, 14], but the classification and nomenclature of the systemic vasculitides have undergone numerous revisions since.

### AAV: changing names and classification

Since the first descriptions by Godman and Churg, vasculitis has been defined by the calibre of the vessel involved, with the term ‘microscopic polyarteritis’ used to distinguish some cases from the earlier descriptions of periarteritis. The American College of Rheumatology first published formal criteria for the classification of vasculitis in 1990 with a reported sensitivity and specificity of 92 and 88 %, respectively for WG, while a definition of MPA and the use of ANCA were not included [15]. These criteria were amended in 1994 by the Chapel Hill Consensus Conference (CHCC) which restricted cases of WG to those with granulomatous inflammation and introduced microscopic polyangiitis as a novel category to cover cases without [16]. Also, the concept of surrogate markers was introduced to aid diagnosis (but not outlined in detail) which included ANCA, although these were not incorporated into formal definitions. Sorensen et al. could not validate the CHCC criteria, finding that only 8/27 patients were accurately classified as having WG and only 3/12 as having MPA [17]. Consequently, they proposed a further amendment, incorporating biopsy findings which were purposefully excluded from the CHCC definitions to facilitate application of the criteria to critically-ill patients in whom tissue sampling may not be feasible. In turn, the Sorensen criteria were shown to perform poorly on a cohort of 99 vasculitis patients with few classified in the MPA category and many excluded from being WG due to eosinophilia [18].

**Fig. 1:**
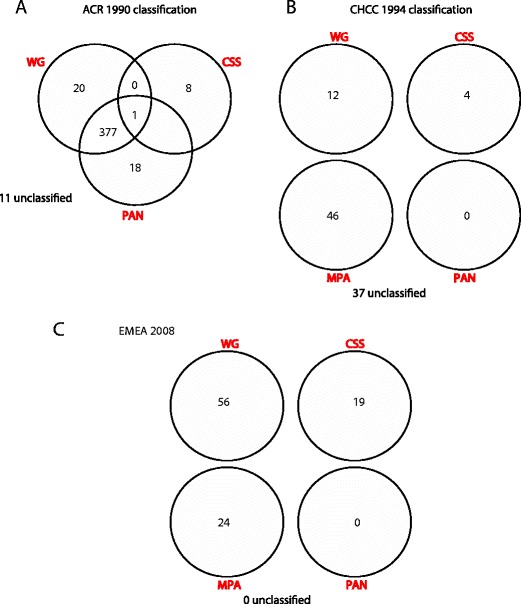
The changing classification of AAV (adapted from Watts et al [20].). A single cohort of 99 patients with AAV (from Lane et al [4].) were assigned diagnostic labels using **a** the American College of Rheumatology 1990 criteria [15], **b** the Chapel Hill Consensus Conference definitions [16] and **c** the EMEA algorithm [20]. *WG* Wegener’s granulomatosis, *CSS* Churg-Strauss syndrome, *MPA* microscopic polyangiitis, *PAN* polyarteritis nodosa

Therefore, by 2004, two principal sets of criteria were in use. The 1990 American College of Rheumatology (ACR) criteria assigned a classification to most patients but did so with an unacceptable degree of overlap whereby a single patient was frequently assigned to more than one diagnostic category (45/99 fitting more than one category in a validation study [18]). Alternatively, the CHCC criteria allocated each patient a single category but left an unacceptable proportion as unclassified (37/99 in a validation study [18]). To resolve this situation, a group of doctors interested in the epidemiology of vasculitis met at the European Medicines Agency in September 2004 and January 2006. They developed a system which, when applied along with the ACR and Lanham criteria [19] to exclude CSS, could accurately resolve all cases to a single diagnosis (Fig. 1) [20]. The European Medicines Agency (EMEA) algorithm is optimally employed at least 3 months after diagnosis, is intended for classification rather than diagnosis of cases and includes, but does not require, biopsy evidence of vasculitis and positive ANCA serology (in practice it is often not possible to obtain biopsy evidence at diagnosis, the inclusion of which risks restricting classification to a subset of research or referral centres). A clear list of surrogate features for use in support of a diagnosis of WG is provided including radiological, histological and clinical markers of granulomatous upper and lower airways disease. To confirm that the accurate classification seen in this description does not derive from overfitting of a classification model to a relatively small cohort of patients, the EMEA algorithm has now been independently validated on a larger cohort of 550 Chinese vasculitis patients [21]. A further change to the nomenclature of AAV occurred in 2012, following a second Chapel Hill Consensus Conference (CHCC 2012). Eponyms were replaced where possible with terms consistent with the known pathophysiology of each condition; hence, Wegener’s Granulomatosis was renamed granulomatosis with polyangiitis (Wegener’s) and Churg-Strauss syndrome was renamed eosinophilic granulomatosis with polyangiitis. ANCA-negative AAV was also recognised where a patient otherwise fulfils the definition for an AAV but has negative results on serologic testing for ANCA. When referring to ANCA-associated vasculitis as AAV, CHCC 2012 also recommended that a prefix should specify ANCA reactivity, i.e. MPO-ANCA, PR3-ANCA or ANCA-negative AAV [22].

### Epidemiology of AAV

The incidence of AAV across different populations is broadly similar at 12–18 per million population per year [23, 24]. The type of vasculitis, however, varies, with GPA being more common in populations from Norway and the UK, and MPA more common in Spain and Japan [23–25]. The prevalence of GPA in a predominantly Northern European Caucasian population from New Zealand was similar to that in the UK and Norway [26]. These population differences in predominant type of AAV may reflect genetic differences or environmental factors. The increased frequency in GPA in populations further away from the equator may reflect differences in vitamin D levels, with decreased sun exposure increasing the risk of developing the disease. Low levels of vitamin D are associated with the risk of developing other autoimmune diseases including rheumatoid arthritis, multiple sclerosis and type 1 diabetes [27]. Other environmental risk factors for AAV have been reported, including infection [28–31], silica [32–34], livestock [32], high solvent exposure [32], asbestos [35], pesticides [36] and, more recently, cocaine use contaminated by the antihelminthic drug levamisole [37].

Ethnic differences may influence both the type and incidence of AAV. Assessment of disease by ethnic distributions in US cohorts suggested GPA is more prevalent in Caucasians than African Americans [38, 39]. In New Zealand in 2003, the 5-year incidence of GPA was twice as high in individuals of European ancestry than in those of New Zealand Maori or Asian background, whereas Pacific Islanders had a rate approximately half that of New Zealand Maori or Asian [40]. In a French multi-ethnic population, AAV was twice as common in individuals of European, compared with non-European, ancestry [41], suggesting different degrees of genetic risk.

### Genetics, GWAS and reclassification

Reports of multiple family members affected by AAV also point to genetic risk factors [42, 43]. This degree of risk in GPA was quantified in a familial aggregation study, which found a relative risk of 1.56 for first degree relatives of GPA [44], similar to that seen in rheumatoid arthritis [45, 46]. A number of candidate gene association studies have been published implicating a number of genetic variants, both single nucleotide polymorphisms (SNPs) and copy number variants (CNVs) in AAV risk. However, many of these were small and unreplicated [47–49].

The European Vasculitis Genetics Consortium recently set out to investigate these genetic risk factors further [50]. Firstly, a genome-wide association study (GWAS) was performed in a discovery cohort of 1,233 UK patients with AAV (MPA and GPA only) and 5,884 UK controls. In the second phase of this project, single nucleotide polymorphisms (SNPs) that significantly associated with AAV were genotyped in a replication cohort of 1,454 Northern European patients and 1,666 controls. In addition, a number of SNPs which had previously been associated with AAV in candidate gene studies were examined, even where they had not reached significance in the discovery cohort, or if they were not represented on the platform used for the GWAS. Several genetic loci were significantly associated with AAV, although on subgroup analysis different associations were identified with GPA and MPA [50].

### Genetic associations within the major histocompatibility complex

The human major histocompatibility complex (MHC) region spans 3.6 Mb containing more than 250 genes, more than half of which have immunological functions. Variants in MHC have been strongly associated with several autoimmune diseases, including type 1 diabetes [51] and SLE [52]. Prior to the GWAS, GPA had been associated with an allele encoding Class II molecule DPB1*0401 (odds ratio (OR) of 3.91, *p* = 1.51 × 10^−10^). In addition, an extended haplotype DPB1*0401/RXRB03 showed an even stronger association with GPA (OR 6.41, *p* = 7.13 × 10^−17^) [53], raising the possibility of additional susceptibility loci. This association of the DPB1*0401 allele with GPA was replicated by a second study, which found a SNP 3′ of *HLA-DPB1* was most associated (*p* = 6.4 × 10^−8^), whilst another SNP in the vicinity of *RING1* (which encodes a transcriptional repressor) also showed a significant GPA association that was partly independent of the *HLA-DPB1* effect [54].

The GWAS confirmed the association of AAV with three SNPs in the MHC. The most significant of these was a SNP in the gene encoding HLA-DPB1, rs 3117242, (OR 3.67, *p* value 1.5 × 10^−71^). Stepwise logistic regression analysis seeking effects that were independent of this SNP failed to find additional distinct susceptibility loci within the MHC [50]. However, when the data were reanalysed by subgroup, the association of HLA-DP was strongest when patients with PR3-ANCA were compared to control (OR 7.03, *p* value 6.2 × 10^−89^). In contrast, although the association between this HLA locus and MPO-ANCA was much weaker (OR 1.55, *p* value 3.2 × 10^−2^), MPO-ANCA was associated with a different HLA gene, *HLA-DQ* (OR 0.65, *p* value 2.1 × 10^−8^). Two previous candidate gene studies have identified an association between susceptibility to eGPA and *HLA-DRB4*, whilst *HLA-DRB3* was associated with protection against the disease [55, 56]. Thus, different HLA genes confer varying degrees of genetic risk across the three subtypes of AAV. A further GWAS analysis of 492 GPA patients and 1,506 healthy controls performed by the Vasculitis Clinical Research Consortium (VCRC) also identified association with HLA-DP, replicating in a further cohort of 528 cases and 1,228 controls [57].

### Genetic associations outside the major histocompatibility complex

The quantile-quantile plot generated by the GWAS showed deviation from the null distribution at the extreme end, even when SNPs in the MHC region were removed, thus non-MHC-linked loci also contribute to AAV susceptibility. Previous studies suggested that variants in the genes encoding proteinase3 and alpha 1 anti-trypsin confer risk for AAV.

PR3 is released when activated neutrophils degranulate, and directly damages endothelial cells—in vitro, the enzyme causes detachment and cytolysis of endothelial cells. The cationic enzyme also binds covalently to the endothelium, where it can bind ANCA and thus trigger antibody-dependent cytotoxicity [58]. Alpha 1 anti-trypsin (α-1-AT) is the major inhibitor of PR3 activity, and is thought to limit the damage done to local tissues. The gene encoding α-1-AT (*SERPINA1*) is highly polymorphic, and several of the polymorphisms, including the Z and S alleles, reduce the function of the protein. The decreased function of α-1-AT may result in persistence of PR3 in inflammatory tissue for longer, which may in turn result in the generation of ANCA. Candidate gene studies showed an association of the Z and S allele with cANCA- and pANCA-positive vasculitis, respectively [56, 59], anti-PR3 antibodies [60], GPA [61, 62] or all AAV [63, 64]. Neither anti-PR3 antibodies nor clinical features of GPA were detected in 191 α-1-AT Z homozygotes [65], confirming that deficiency in α-1-AT is not enough by itself to cause GPA.

In the GWAS, the strongest association with AAV outside the MHC was found at rs 7151526 in the *SERPINA1* locus (OR 0.59, *p* value 2.4 × 10^−9^). This association was strongest when anti-PR3 was compared with control (OR 0.53, *p* value 5.6 × 10^−10^), but was not significant when anti-MPO was compared with control [50]. The VCRC GWAS also identified an association with semaphorin 6A (SEMA6A), although this achieved genome-wide significance in the combined cohorts used rather than demonstrating independent replication [57].

PR3 is expressed on the surface expression of neutrophils in a bimodal pattern, with the proportion of neutrophils that are high or low expressers remaining constant over time, and unaffected by age or gender [66]. This surface expression pattern correlates much more closely in monozygotic (*r* = 0.99) than dizygotic twins (*r* = 0.06), suggesting that it is genetically determined [67]. Patients with AAV have an elevated level of membrane bound PR3 expression, and this is associated with an increased relapse rate [68, 69]. The gene encoding PR3, *PRTN3*, was not well represented on the platform used for the GWAS, but a previous candidate gene study had shown an association between a polymorphism in the promoter region of *PRTN3*, affecting a putative transcription factor binding site, and GPA [70]. Thus, a SNP in PRTN3, rs 62132295, was genotyped by Lyons et al in the second phase of their study, and was significantly associated with PR3-ANCA (OR 0.73, *p* value 2.6 × 10^−7^) but not MPO-ANCA (OR 1.1, *p* value 2.2 × 10^−1^) [50].

### Anti-MPO and anti-PR3 AAV as genetically distinct diseases

The most striking finding of the GWAS and replication studies was the disparity between the genetic associations when GPA and MPA were analysed separately. Patients were classified with GPA or MPA according to the EMEA algorithm described previously [20] and, in addition, patients had to have either an MPO- or PR3-ANCA, or a diagnostic tissue biopsy with cANCA or pANCA. For the subgroup analysis, genetic associations were compared with the diagnoses of GPA or MPA, as well as with PR3- and MPO-ANCA. As described above, a SNP in the HLA-DP locus was more strongly associated with PR3-ANCA than GPA, but showed no association with MPO-ANCA. The same pattern of association was seen with SNPs in *SERPINA1* and *PRTN3*, as well as other SNPs in other loci e.g. the gene encoding Rho GTPase-activating protein 18 (*ARHGAP18*) and the gene encoding motile sperm domain-containing protein 2 (*MOSPD2*). One SNP in HLA-DQ was more significantly associated with MPO-ANCA compared with PR3-ANCA. However, in the cohorts of patients recruited to this study, only 489 had MPA (556 had MPO-ANCA) compared with 1,683 with GPA (1,521 with PR3-ANCA). This reduced the power of the subgroup analysis to detect associations with MPO-ANCA in particular [50]. Nevertheless, it is clear that there are different genetic contributions to AAV associated with PR3-ANCA to that associated with MPO-ANCA, and further studies are planned to address the genetics of these two conditions separately which may identify further disease specific genetic susceptibility loci. There may also be implications for clinical studies that analyse treatment outcomes for GPA and MPA together, as such genetically distinct conditions might be expected to have different clinical courses.

It should be noted that the platforms used for GWAS cover at best 97 % of the genome, and areas where coverage is poor are those where there are segmental duplications and extensive copy number variation [71]. Thus, there may be important genetic associations with AAV that were not been detected in the GWAS. For example, previous candidate gene studies have identified associations with polymorphisms in the genes encoding Fcγ receptors (for immunoglobulin G) [49], but this region is very poorly covered by SNP array platforms.

## Histopathology of AAV

Histologically, AAV is a necrotising vasculitis with few or no immune deposits predominantly affecting small vessels (i.e. capillaries, venules, arterioles and small arteries). Histology is also critical to the CHCC classification of AAV: in MPA, necrotising arteritis, glomerulonephritis and capillaritis are common, but granulomatous inflammation is absent. GPA is characterised by necrotising granulomatous inflammation involving the upper and lower respiratory tract. Necrotising glomerulonephritis, pulmonary haemorrhage and ocular vasculitis are also frequently seen in GPA. In eGPA, the necrotising granulomatous inflammation is eosinophil-rich and frequently involves the respiratory tract, with ENT involvement often manifest as nasal polyps [22].

All three subtypes of AAV may cause a necrotising glomerulonephritis, although this is less common in eGPA, particularly ANCA-negative eGPA [22]. Renal involvement is associated with higher morbidity and mortality [72]. A histopathological classification scheme has recently been described that can prognosticate renal survival [73]. One hundred renal biopsies were assigned to one of four categories:

1. Focal. >50 % normal glomeruli
2. Crescentic. >50 % glomeruli with cellular crescents
3. Mixed. <50 % normal, <50 % crescentic, <50 % globally sclerotic glomeruli
4. Sclerotic >50 % globally sclerotic glomeruli

These categories correlated significantly with renal function at presentation and after 1 and 5 years of follow up. Renal survival (without end-stage renal disease) at 5 years was 93 % for the focal class, 76 % for the crescentic class, 61 % in the mixed category and 50 % in the sclerotic category [73]. Although this classification requires further validation, it may be a useful tool in predicting renal survival at the time of diagnosis.

## The role of ANCA in AAV pathogenesis

**Fig. 2:**
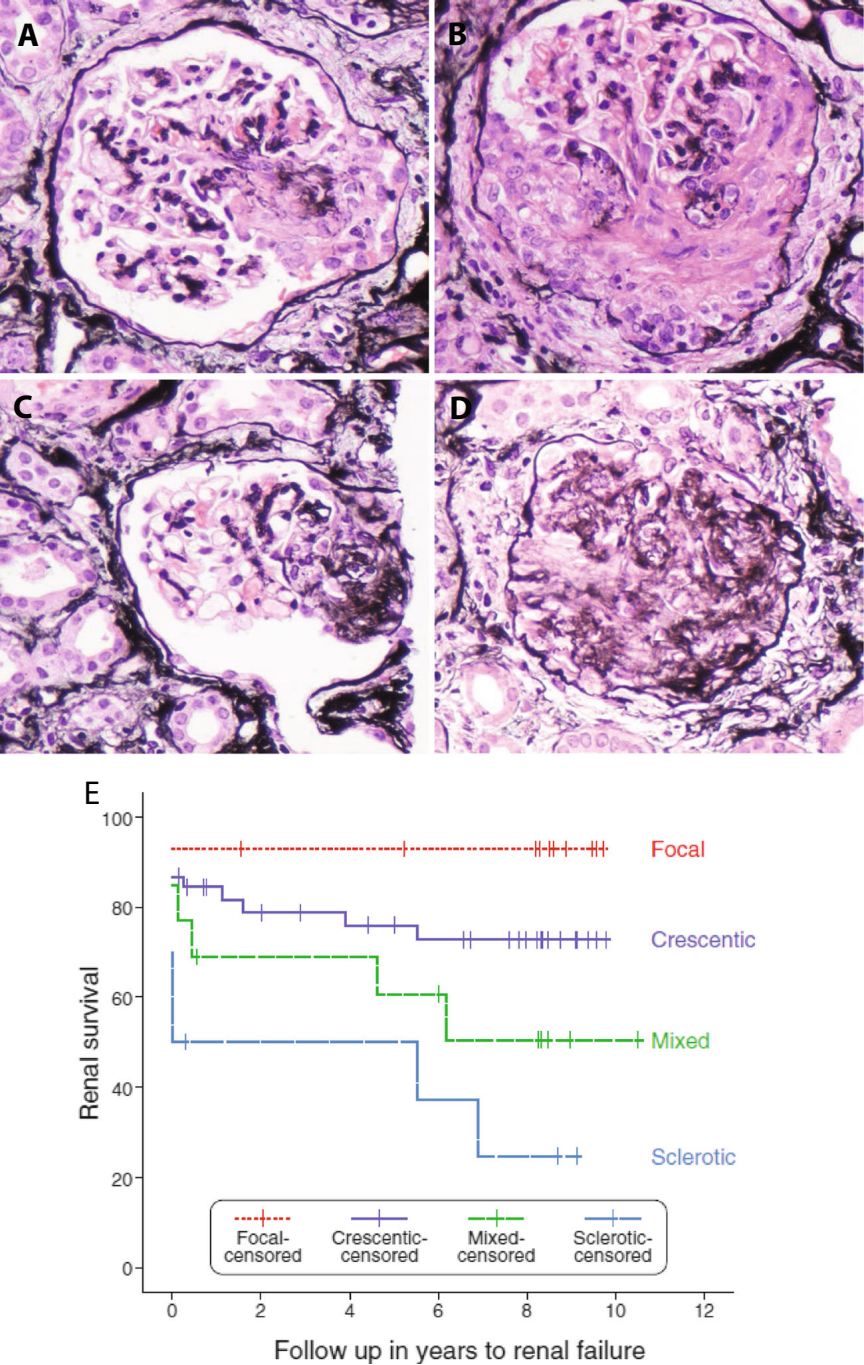
Histopathology of AAV. All pictures show glomerular lesions from one patient with an ANCA-associated vasculitis. (Jones-Methamine-Silver stain, original magnification ×400) **a** Cellular crescent involving <50 % of the glomerular tuft defined (according to the histopathologic criteria for AAV [73]) as a lesion filling the Bowman’s space containing a cellular component of >10 %, regardless of the proportion of the glomerular tuft that is involved (<50 % in this picture). The amount of fibrin or fibrosis does not affect the classification, as long as the required cellular component is seen. **b** Cellular crescent involving >50 % of the glomerular tuft. A case is classified as “crescentic” if ≥50 % of all glomeruli in the biopsy show crescents (regardless of the proportion involved in the individual glomerulus). If <50 % of all glomeruli show crescents, the classification depends on the proportion of normal and sclerotic glomeruli present in the biopsy: any biopsy with ≥50 % normal glomeruli is designated as “focal”. In contrast, any biopsy showing ≥50 % of sclerotic glomeruli is classified as “sclerotic” and a biopsy not fulfilling any of the aforementioned criteria is called “mixed”. **c** Glomerulus with a fibrous crescent defined as a lesion filling the Bowman’s space containing a cellular component of <10 %, affecting up to 80 % of the tuft. The lesion is largely composed of fibrous tissue. **d** Globally sclerosed glomerulus, >80 % of the tuft is sclerotic. This is not specific for AAV. **e** Renal survival (the absence of end-stage renal failure) in a cohort of 82 patients with AAV by histological classification (from Berden et al.) [73]. Inclusion in the classification schema requires a pauci-immune staining pattern on immunofluorescence microscopy and at least one glomerulus with necrotising or crescentic glomerulonephritis on light microscopy

Since the initial observation that circulating ANCA characterise the condition [74], much debate has centred on whether and to what extent these autoantibodies might play a role in disease pathogenesis (Fig. 2).

### In vitro evidence

Both MPO and PR3 are typically retained within cytoplasmic azurophilic neutrophil granules. However, a small proportion of each is expressed on the neutrophil surface [75] and this is increased in response to TNFα [76, 77], during apoptosis [78] and on exposure to ANCA-IgG [76, 77] although expression is also seen on the surface of monocytes [79].

By contrast with immune complex disorders, AAV is characterised by so-called ‘pauci-immune’ vascular inflammation, in which immunoglobulin deposition cannot be detected, and the proposed role for ANCA is therefore not of direct toxicity and tissue damage but of indirect recruitment of an autoinflammatory response. Neutrophils may be activated in vitro by ANCA-IgG but only when primed with TNFα [76, 77] in a complex process shown to involve both FcγRIIa-dependent [80, 81] and independent [82–84] mechanisms. ANCA-binding may therefore encourage further neutrophil expression of the autoantigens MPO and PR3, perpetuating the antigen-specific response. MPO and PR3 also directly bind to endothelial cells causing direct apoptosis [85] in addition to that caused by ANCA-activated neutrophils [86] in an integrin-dependent fashion [87]. Thus, a pathogenic model has been proposed whereby, in the presence of accessory inflammatory signals (such as LPS or TNFα), neutrophils may be further stimulated by circulating ANCA to cause endothelial toxicity, necrosis and organ dysfunction [88].

### In vivo evidence

High circulating titres of ANCA are typically seen during active disease with subsequent falls during therapy, while drug-induced forms are accompanied by the appearance of ANCA which resolve on treatment and removal of the provoking agent [89]. These observations may be consistent with either a pathogenic role for ANCA but also with the possibility that ANCA represent an epiphenomenon, accompanying but not necessarily driving disease. ANCA are not detectable in approximately 20 % of cases, clinical benefit following B cell-depleting rituximab therapy is often seen before a significant reduction in ANCA titre [90] and has also been reported in ANCA-negative cases [91].

Two reports of newborns who have developed glomerulonephritis and lung haemorrhage within days of delivery to mothers with circulating anti-MPO antibodies and active vasculitis, and responded rapidly to treatment with plasma exchange, provide direct evidence of ANCA-related pathogenicity in humans [92]. Animal models also support a pathogenic role for ANCA. Rats immunised with human MPO develop anti-MPO antibodies that cross-react with human and rat MPO, and thus generate a small-vessel vasculitis. Passive transfer of IgG from these rats into naive rats also induced disease [93]. Mice do not develop an immune response to human MPO, but MPO-deficient mice injected with mouse MPO do generate an immune response, such that injection of anti-MPO IgG from these mice into wild type mice results in a pauci-immune glomerulonephritis and small vessel vasculitis comparable to human AAV disease [94]. Transfer of anti-MPO IgG into Rag2^−/−^ mice (that lack functioning lymphocytes) also causes disease, but pre-treatment of wild type mice with a neutrophil-depleting monoclonal antibody is protective, suggesting neutrophils, but not lymphocytes, are the main drivers of disease in this model [95]. In addition, transfer of a T cell-enriched (>99 %) splenocyte fraction from MPO-immunised MPO^−/−^ mice did not reproduce the vasculitis seen after unfractionated splenocyte transfer (a preparation comprising approx 25/65 % T/B cells, respectively [88]). However, this does not preclude a role for T cells in disease pathogenesis. The proportion of glomeruli showing necrotic changes was substantially higher in the splenocyte transfer compared to the passive Ig transfer model (80 vs 15 % crescent formation) although the disease was not strictly pauci-immune, demonstrating evidence of immune complex deposition, albeit to a similar degree seen in control (non MPO-immunised MPO^−/−^) splenocyte transfer experiments [94].

These studies support a pathogenic role for MPO-ANCA in AAV. In vivo evidence for anti-PR3 antibodies in disease causation is less convincing, however. Similar experiments where PR3-deficient mice are immunised with murine PR3, then anti-PR3 IgG from these mice is transferred into wild type mice, does not result in vasculitis of the lung or kidney, although intravenous injection of anti-PR3 IgG does enhance panniculitis at sites of subcutaneous TNFα injection [96]. More recently, this model has been enhanced by using an LPS-primed ‘humanised’ mouse model (irraditated NOD-scid-IL2Rγ^−/−^ reconstituted with human haematopoietic cells) in which passive transfer of PR3-IgG ANCA from patients did result in a pauci-immune glomerulonephritis and alveolar haemorrhage [93].

Additionally, development of disease may require the presence of a ‘susceptible’ genetic background in addition to ANCA. The transfer of splenocytes from the autoimmune-prone NOD mouse after immunisation with recombinant murine PR3 (rmPR3) generated a necrotising glomerulonephritis in immunodeficient NOD-SCID recipients but this was not seen after similar transfer of splenocytes from rmPR3-immunised C57BL/6 control mice, implicating the autoimmune-prone background as a critical factor in expression of a disease phenotype [97].

This proposal could account for observations in humans that healthy individuals may have ANCA [98] with or without T cells reactive to either PR3 or MPO antigens [99] without developing disease.

### Complement and AAV

Surprisingly, although deposits of ANCA and immune complexes are not seen in pauci-immune vasculitic lesions, complement may nonetheless play an important role in pathogenesis. Adoptive transfer of anti-MPO antibodies can induce a crescentic glomerulonephritis in recipient mice [94] but disease is prevented if recipient mice are deficient in components of the alternative (C5, factor B) but not classical (C4) complement pathway [100] and can be treated (in mice) by blocking the terminal complement component C5 [101]. In small cohorts of human AAV patients, alternative complement components (factor B, properdin) have been reported in inflamed vessels [102, 103] with corresponding plasma levels similarly suggesting alternative pathway activation [104]. It has been suggested that the higher levels of C5a seen in active AAV may contribute directly to neutrophil priming as part of an ‘amplification loop’ [105, 106]. The importance of C5 signalling has been shown both in vitro, as C5-stimulated neutrophils release further C5a [105], and in vivo as C5aR-deficient mice fail to develop disease in MPO transfer models [106].

### Models of broken tolerance in AAV

While ANCA may play a role in driving vasculitis, initial events leading to a breakdown of tolerance and generation of autoantibodies have remained unclear. There is increasing evidence to support the role of bacterial infections as the initial event that breaks tolerance, resulting in autoimmunity.

Three key hypotheses currently dominate the field.

**Fig. 3:**
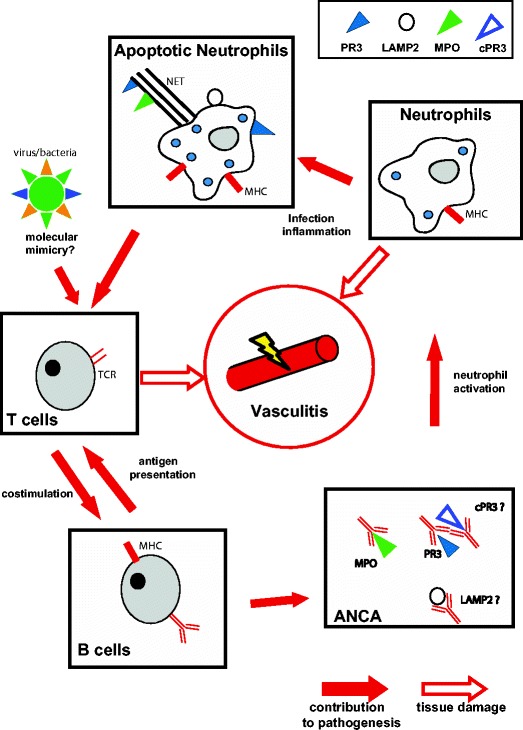
Pathogenesis of AAV. Schematic illustration of our current understanding of the pathogenesis of AAV. *TCR* T cell receptor, *PR3* proteinase-3, *MPO* myeloperoxidase, *LAMP-2* lysosome-associated membrane protein-2, *rPR3* reverse PR3

1. Anti-idiotype antibodies and complementary PR3
  An antibody may recognise the complementarity-determining region (CDR) of another antibody in what has been termed an anti-idiotype response [107]. While investigating PR3 peptides inserted into a bacterial artificial chromosome expression system, Falk and colleagues noted that GPA patient serum reacted to a PR3 peptide which had been inadvertently inserted in reverse. They postulated that a pathological anti-PR3 response may develop as an anti-idiotype response to such a complementary PR3 (cPR3) protein. They subsequently showed that GPA patients with PR3-ANCA also had antibodies that could react with this in vitro-generated cPR3 peptide (and that anti-PR3 could be induced in naïve mice by immunisation with anti-cPR3 antibody) [108]. However, these data have not been confirmed in other patient cohorts [109] and, although widespread sense-antisense gene pairs have been reported [110], there is as yet no evidence for in vivo translation of the cPR3 sequence into peptide. Further research is therefore necessary before the concept of an anti-idiotypic antibody network can be accepted as playing a role in the pathogenesis of AAV. Indeed, even if this proves to be the case, cPR3 would still be an endogenous ‘self’ peptide and an explanation for broken tolerance must still be sought. The peptide sequence of cPR3 has been shown to have at least partial homology with sequences found in a range of microbes [108], including *Staphylococcus aureus*. However, there is no evidence that anti-cPR3 antibodies cross-react with *S. aureus.* Furthermore, rodents immunised with *S. aureus* do not develop AAV [111], though interestingly immunisation with *Escherichia coli* used as a control in this study did induce AAV in a small proportion of animals. *S. Aureus* incidence is, however, higher in GPA patients compared to healthy controls, colonisation is associated with increased relapse rates (relative risk 9.0) [112] and treatment with cotrimoxazole (aiming to eradicate the bacteria) can reduce relapse rates [113].
2. Anti-LAMP antibodies and molecular mimicry
  An alternative hypothesis has been proposed, founded on principles of molecular mimicry. By probing membrane protein fractions isolated from human neutrophils and glomeruli with sera from ANCA patients, the protein lysosome-associated membrane protein 2 (LAMP2) was identified as a possible autoantigen [114]. LAMP2 is a shuttle protein carrying complex oligosaccharides from lysosomal compartments to the cell surface in neutrophils, amongst other cell types, and also plays a role in intercellular adhesion, through effects on the expression of E-selectin [115]. Kain et al. showed that circulating anti-LAMP2 antibodies were present in 78/84 AAV patients with active glomerulonephritis irrespective of other ANCA specificities (84 IF ANCA^+^, 38 MPO^+^, 39 PR3^+^) and that autoantibody levels fell with recovery. Intriguingly, they also demonstrated that rabbit anti-human LAMP2-IgG (which cross-reacts with rat LAMP2) could induce a pauci-immune glomerulonephritis on passive antibody transfer into Wistar Kyoto (WKY) rats and that LAMP2-IgG could activate primed neutrophils and cause apoptosis in cultured endothelial cells in much the same way as the ‘classical’ ANCA MPO- and PR3-IgG had been shown to do [31]. Furthermore, while LAMP2 showed no homology with MPO or PR3 (thus excluding synergy with the anti-idiotype hypothesis), it showed significant similarity to the sequence of the bacterial protein FimH, which could induce both LAMP2-IgG and a pauci-immune glomerulonephritis on immunisation of WKY rats.
  Therefore, infection with a fimbriated FimH-expressing organism such as *E. coli* or *Klebsiella pneumoniae* could feasibly lead to development of a pathological anti-LAMP2 response through molecular mimicry, although this hypothesis also requires further confirmation, as detection of anti-LAMP2 antibodies in independent cohorts has been hampered by technical difficulties with the assay [116]. While molecular mimicry by bacterial pathogens may contribute to the development of vasculitis, the prevalence of such microbial infections and the comparative rarity of systemic vasculitis indicate that an inherent susceptibility to the breakdown of tolerance must also be required. It should be noted, however, that the presence of circulating anti-LAMP2 antibodies remains controversial as, although the original group have replicated their findings [117], other groups have not been able to do so [118].
3. NETosis
  Neutrophil extracellular traps (NETs) are structures of chromatin fibres released by dying neutrophils that trap and kill extracellular invading microbes [119]. However, this glutinous DNA web, which contains a number of antimicrobial peptides including MPO and PR3, can also stick to and damage the endothelium of small blood vessels, causing vasculitis [120, 121]. Even in the absence of infection, a greater proportion of neutrophils incubated with purified IgG from patients with AAV formed NETs compared with neutrophils incubated with serum from healthy controls. Components of NETs were identified in close proximity to neutrophils seen in renal biopsy specimens from individuals with AAV and acute deterioration of renal function [120]. In vivo, myeloid dendritic cells uploaded with and activated by NET components induce ANCA and autoimmunity when injected into native mice [121].
*S. aureus* is known to strongly induce NETs [122]. Thus, the propensity of patients with AAV to form NETs may be exacerbated by infections with this bacterium, which could therefore explain the relationship between *S. aureus* and increased relapse risk described above (Fig. 3).

### T cells in AAV

Tracking the emergence and progression of histological changes taking place in relatively inaccessible sites, such as the respiratory tract, is further complicated by the often insidious presentation of the disease resulting in late diagnosis. This is compounded by the lack of an appropriate animal model—none of the currently available models develop the characteristic granulomatous changes of GPA. Despite these limitations, there is strong evidence to support a key role for T cells in the aetiopathogenesis of AAV.

### T cells in histology

It has been proposed that a localised form of GPA may begin with extravascular granuloma formation [123]. Biopsy series suggest that early foci of tissue injury are followed by a mixed inflammatory cell infiltrate including neutrophils, monocytes, lymphocytes and, later, epitheloid and multinucleate giant cells. Mononuclear histiocytes have been observed to surround (or ‘palisade’) a central area of necrosis with the development of neutrophilic microabscesses. Such lesions are highly heterogeneous and may be found either adjacent to blood vessels or at more distant sites [123, 124]. The apparent early development of these lesions has been suggested to occur in response to unknown, possibly infectious, immunogens resulting in a localised phase of disease before tolerance is broken and systemic manifestations develop. It has long been appreciated that the infiltration of lymphocytes, particularly CD3^+^ T cells, is characteristic of these lesions [125] which show substantial structural organisation. Myeloid cells (monocytes and neutrophils) expressing high levels of the PR3 autoantigen [126] arrange in clusters and are surrounded by activated APCs [127], B cells and T cells expressing markers of effector differentiation [128]. The development of germinal centre-like structures has been observed [129] with some evidence for T cell-mediated B cell maturation occurring [130]. The predominance of the isotype-switched IgG_1_ and IgG_4_ subclasses amongst circulating ANCA is further evidence of T cell help contributing to the pathological response [131].

### Circulating T cells

Measurements of CD4 and CD8 T cell subsets have consistently shown an overall lymphopaenia in AAV [132, 133] with a reduction in the CD4 to CD8 ratio. It remains unclear, however, whether this is driven by disease per se or may be related to concurrent immunosuppressive therapy and whether it reflects a relative reduction in CD4 counts or an expansion of CD8 cells. A single, small study suggesting similar changes prior to therapy [134] and lymphopaenia beyond that seen in other immunosuppressed cohorts (such as renal transplant recipients) [132], together imply that it may be a disease-related phenomenon.

Studies of surface marker expression on circulating CD4 T cells have identified increased expression of costimulatory molecules and markers of activation including HLA-DR, CD137 [135] and CD25 [136, 137] which could not be explained by altered numbers of the regulatory CD4 subset (CD4^+^25^+^FoxP3^+^) [132]. Increased CD25 expression was seen predominantly on CD4 cells with a naïve phenotype (CD4^+^CD45RO^−^ [132, 137]), suggesting that these changes might reflect an inherent increased expression of the activation marker rather than increased expression due to an activated phenotype. Associations of polymorphic variants in the *IL2RA* gene which encodes CD25 with vasculitis further support this possibility [138, 139]. While several groups have shown no change in numbers of circulating regulatory T cells [132, 137, 140], others have shown this population to be increased but with diminished suppressive function in in vitro co-culture assays with autologous stimulated T cells [133]. Possible explanations for these contrasting findings include the use of FoxP3 expression to define a regulatory T cell population, as this transcription factor can be transiently upregulated following human T cell activation [141]. Furthermore, these small studies incorporated patients with variable levels of disease activity and therapy at the time of sampling. The association of increased CD25 expression is a more robust finding, with increased levels of the soluble form of CD25 also correlating with disease activity [142, 143]. An increased circulating Th17 subset (IL-4^−^ IL-17^+^) has also been reported in the remission phase [144].

Observations by Kallenberg and colleagues that AAV patients have increased levels of circulating CD4 T_EM_ cells (CD4^+^CD45RO^+^CCR7^−^) during remission phase are also consistent with persistent activation of circulating T cells in AAV [128, 140]. In one study, a significant decrease in the circulating proportion of such cells during active renal disease and their concurrent appearance in urine was shown, consistent with known migratory patterns of effector T cells into tissues and also with a role for these cells in contributing to the tissue damage seen [145].

Studies of altered CD8 T cell phenotypic markers in AAV are less common. One study identified a population of CD8^+^CD11b^+^CD28^−^ cells which was expanded in AAV and more so in those patients with longer disease duration (>5 years), although this expansion was not linked to prognosis and showed positive correlation with increasing age [146].

### T cell function in AAV

Several studies have attempted to identify underlying differences in T cell responses following in vitro stimulation with ANCA autoantigens. The direct lymphocyte toxicity of the Triton-X100 detergent used commercially to isolate proteinase 3 from neutrophil azurophilic granules [147] precludes its use for stimulation studies and confounded some early reports [148, 149]. Others have attempted to bypass this problem using detergent-free methods. However, these have not achieved equivalent purities [150], often with persistent contamination with MPO or other granule contents [151]. The largest study to date assessed in vitro proliferation of PBMC cultured with heat-inactivated or native PR3 and MPO in 45 AAV patients at various stages of activity and treatment (16 receiving no immunosuppression). In this study, T cells from patients showed a significant increase in proliferation compared to healthy controls, although this effect was largely driven by the responses of a subset of ~20 % of patients, was seen to be dampened by concurrent therapy, was only seen with heat-inactivated PR3 (i.e. not with either native PR3 or MPO) and the T cells of healthy individuals also showed proliferative responses albeit to a lesser degree [150]. Smaller studies (*n* = 15–21) including patients in remission on no therapy [152] or during active disease with therapy [153, 154] have shown proliferative responses to native PR3 and MPO, again in only a subset of patients and again similar proliferative responses were seen in either healthy or diseased controls (with either Goodpasture’s disease or IgA nephropathy).

### TCR repertoire studies in AAV

An alternative approach to assessing T cell involvement in AAV has been to profile the T cell receptor (TCR) repertoire of circulating T cells with the hypothesis that over-representation of T cells bearing a particular TCR variable region may indicate responses to a common triggering antigen. One study identified clonal expansion (by restricted CDR3 length) of a particular Vβ segment (BV8) in circulating CD4 T cells in four of eight patients that were not seen in either healthy or disease controls (with RA) [155]. A larger study of 28 patients with a mixture of MPA and GPA found over-representation of a Vβ2.1 junctional region sequence in MPA but not in GPA patients or healthy controls. This was seen to occur in a polyclonal pattern which was interpreted by the authors as possible evidence of a superantigen effect in which all Vβ2.1 TCR-bearing T cells may be non-specifically stimulated by bacterial antigen such as those seen in *S. aureus* [156]. While *S. aureus* carriage is a known risk factor for relapse in GPA [112], a further, larger study confirmed the presence of specific T cell expansions in GPA patients but no link with those predicted to bind staphylococcal superantigens was seen [157].

Strong evidence therefore implicates both CD4 and CD8 T cells in the pathogenesis of AAV. They are present within disease lesions, closely apposed to both other T cells, APCs and B cells to which they are likely to provide help in generating the isotype-switched high-affinity autoantibodies which characterise the disease.

### Treatment and outcome

The introduction of immunosuppressive therapy has altered the prognosis of vasculitis, changing it from a frequently fatal condition (with 1 year untreated mortality rates of approximately 80 % [158]) to a chronic relapsing and remitting disease. Five year survival rates are around 75 %, with 1 year survival rates of 84 % [159, 160]. Early predictors of mortality include infection, leukopenia and cumulative cyclophosphamide dose [161], hence infectious complications of immunosuppressive therapy now contribute more to morbidity and mortality than complications of the disease itself [159, 162].

**Fig. 4:**
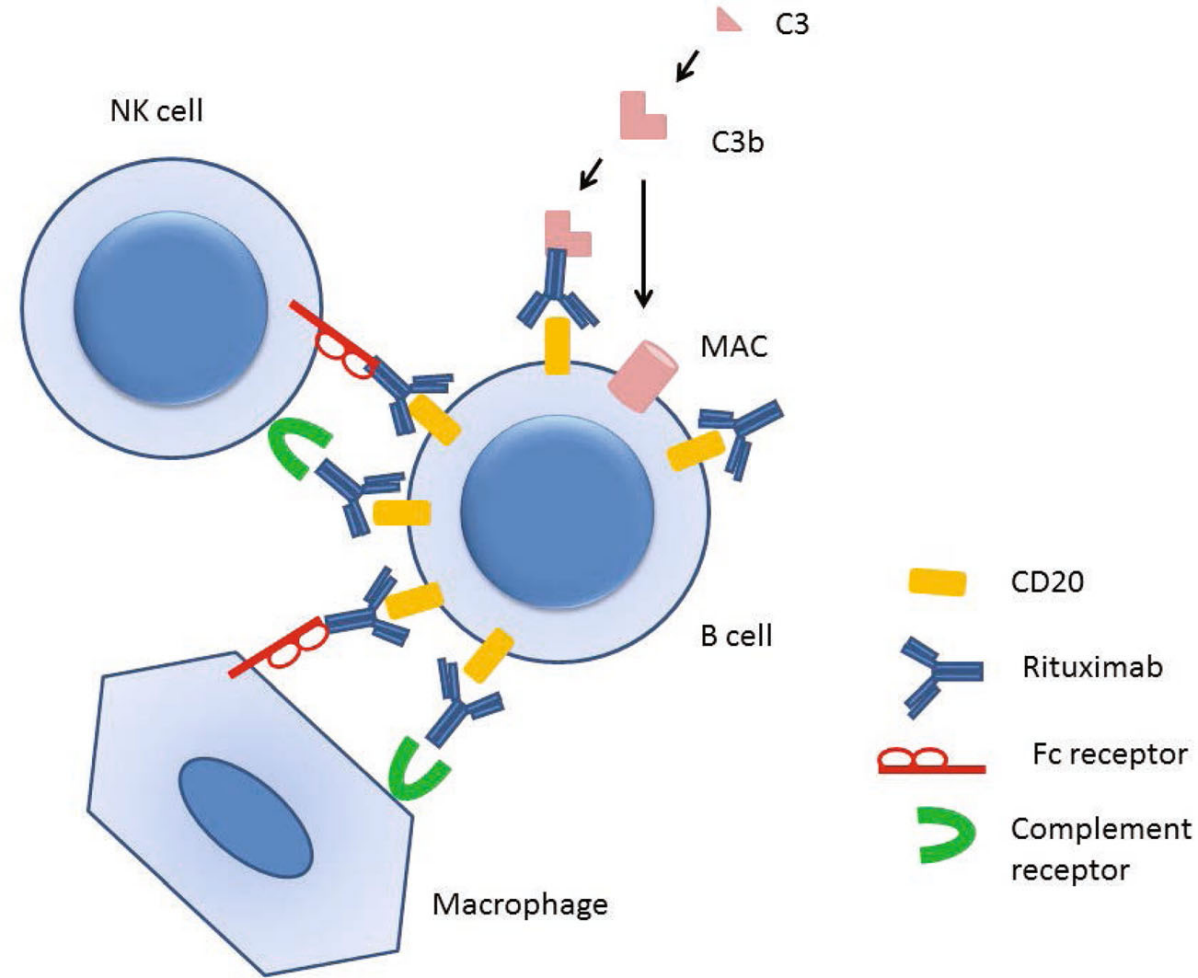
Mechanisms of B cell depletion with rituximab therapy

Although therapy successfully induces remission in up to 90 % of survivors [160], 38 % of patients will relapse within 5 years, with GPA being associated with a much higher risk of relapse than MPA [163]. New immunosuppressive treatments, such as the depleting monoclonal antibody rituximab, promise to allow induction and maintenance of remission [90] while limiting infectious complications [164]. Rituximab depletes CD20^+^ B cells by antibody-dependent cellular cytotoxicity, complement-dependent cytotoxicity and apoptosis in an Fcγ receptor-dependent fashion [165] (Fig. 4). Rituximab was originally developed to treat non-Hodgkin’s lymphoma, for which it was licensed by the FDA in 1997, but was then shown to be an effective treatment for rheumatoid arthritis and later for AAV also [90]. Two recent randomised controlled trials in patients with GPA and MPA found that rituximab was as effective an induction agent as cyclophosphamide, but in these studies there was no difference in infection rates. In the rituximab for ANCA-associated vasculitis (RAVE) study, which recruited relapsing as well as new AAV, rituximab was more effective at inducing remission in relapsing disease [166] and this superiority persisted at 12 months [167]. Relapse rates were not significantly different following rituximab induction therapy with no ongoing maintenance treatment, or induction with daily oral cyclophosphamide followed by azathioprine maintenance treatment—in both groups, approximately 30 % of patients had relapsed by 18 months [167]. Retrospective data supports the use of repeated dosing with rituximab every 6 months for 2 years to maintain remission, with relapse rates of only 12 % during 24 months of treatment [168]. There is, however, a risk of hypogammaglobulinaemia associated with long term treatment with rituximab [169]. Randomised studies of rituximab versus azathioprine as maintenance therapy are ongoing.

Given the data presented above on the role of T cells in the pathogenesis, there is a rationale to target T cells, or at least T cell help, to treat AAV (an often-overlooked result of B cell depletion also). Alemtuzumab is an anti-CD52 monoclonal antibody that depletes mature lymphocytes. It has been used to treat refractory AAV—a retrospective series from Cambridge UK described 71 patients with relapsing or refractory disease, of whom 85 % achieved remission following treatment with alemtuzumab. However, mortality and morbidity were high—28 patients developed infection, and 31 patients died at a median of 106 months [170]. Nonetheless, the patient in this cohort had previously been heavily immunosuppressed for refractory disease (median cyclophosphamide dose 150 g)—and given this, alemtuzumab remains a potential alternative therapy that warrants further investigation. Another biologic therapy that shows promise in GPA is abatacept. This fusion protein combines the Fc region of IgG1 with the extracellular domain of CTLA, and blocks costimulation of T cells. It is licensed in the USA for treatment of rheumatoid arthritis, and is currently being trialled for lupus nephritis in combination with cyclophosphamide. An open label trial of abatacept in non-severe GPA found that 80 % achieved remission at a median of 1.9 months [171]. Thus, therapy targeted to T cells, as well as B cells, may be effective in AAV, although no randomised trials have yet been done in this area. The morbidity, mortality and toxicity arising from the immunosuppression required to control AAV could be alleviated if biomarkers were available to allow targeting of their use to those with most severe prognoses.

A recent systematic review of the literature was conducted by the EULAR systemic vasculitis task force to identify and assess the quality of outcome measures in vasculitis [72]. The level of disease-related damage (assessed by the Vasculitis Damage Index, VDI) was found to inversely correlate with successful remission (RR 1.53) while, curiously, those with higher levels of disease activity (measured by the Birmingham Vasculitis Activity Score, BVAS, >23) were found to more easily achieve remission (RR 2.94), independent of the intensity of induction therapy.

Relapse was associated with three independent factors, firstly, with the level and duration of induction therapy (<10 g cyclophosphamide within 6 months, RR 2.83, prednisolone dose >20 mg/day for at least 2.75 months, RR 2.41, and the use of trimethoprim/sulfamethoxazole RR 0.32), secondly, with target organ involvement (increased for cardiac, OR 2.87 or renal involvement with CrCl < 60 ml/min, OR 2.94 and also for chronic nasal carriage of *S. aureus*, RR 7.16) and thirdly, with the presence of ANCA. Both the presence of ANCA at diagnosis (RR 2.89) and also a rise in PR3-ANCA titre of more than 4-fold (RR 42.5) were associated with subsequent relapses. This is supported by long term follow up data from four European treatment trials of 535 patients with GPA or MPA followed for a total of 1,804 patient years. This study showed that PR3-ANCA is associated with a higher risk of relapse than MPO-ANCA (HR1.62 (95 % CI 1.39–1.89). This was also seen in the 18-month follow up from the RAVE study comparing rituximab induction therapy with cyclosphosphamide followed by azathioprine, risk factors increasing relapse risk included a diagnosis of GPA, anti-PR3 ANCA and a previous history of relapse. Patients with all three risk factors were at highest risk of relapse (approximately 50 % at 18 months in both treatment groups) [167]. This data suggests that the type of AAV and ANCA should be considered when planning the duration and type of remission [163] therapy, although this has not been addressed by any prospective clinical trials.

### Predicting outcome and targeting therapy in AAV

The development of immunosuppressive therapy has changed AAV from a progressive and often-fatal disease, into one that follows a chronic, relapsing-remitting course. However, prognosis is highly variable and current clinical tests—including ANCA—do not allow prediction of subsequent clinical outcome. We have found that patterns of gene transcription within CD8 T cells, measured in patients presenting at diagnosis before treatment has begun, are able to identify a subgroup of patients at high risk of subsequent relapse [172]. This observation has the potential to at once identify novel pathways for treatment of disease and the patients in whom such treatment is required. Similar patterns have been shown to predict clinical outcome in multiple autoimmune and inflammatory conditions [173] suggesting that factors controlling relapse and outcome are shared between diseases and are independent of the mechanism or self-antigen that breaks tolerance in the first place.

## Summary/conclusions

The ANCA-associated vasculitides are rare disorders with high mortality and morbidity attributable not just to the disease but the toxicity of current treatment regimes. This toxicity is compounded by the long duration of treatment required to minimise relapse risk. Recent research has focussed on ways to reduce this treatment burden, both by better understanding of disease pathogenesis to target immunosuppression more selectively and by identification of biomarkers that identify patients at an increased relapse risk. Over the last 20 years, a number of clinical trials have established treatment pathways for GPA and MPA, with these diseases being treated in a similar fashion. Long term follow up of these studies, however, have shown that relapse risk differs between the two conditions, with GPA, and independently PR3-ANCA, being markers of increased relapse risk. Genetics studies have also highlighted the difference between GPA and MPA, and it is perhaps now time to consider different strategies to treat, and in particular prevent relapse, in the two diseases. The discovery of novel biomarkers that predict relapse risk across multiple autoimmune diseases may further pave the way for personalised medicine, where treatment is tailored to an individual’s specific relapse risk, to minimise treatment toxicity. Clinical trials allowing such flexibility of treatment regimes will be particularly challenging in rare diseases like the ANCA-associated vasculitides, but stratification according to biomarkers, together with a commitment from clinicians to recruit as many patients as possible to multi-centre international clinical trials, may make this possible.
